# Effect of a Comprehensive Health Care Program by Korean Medicine Doctors on Medical Care Utilization for Common Infectious Diseases in Child-Care Centers

**DOI:** 10.1155/2014/781675

**Published:** 2014-09-11

**Authors:** Minjung Park, Jimin Park, Soonman Kwon

**Affiliations:** ^1^Graduate School of Public Health, Seoul National University, Sillim-dong, Kwanak-gu, Seoul 151-742, Republic of Korea; ^2^Division of Traditional Korean Medicine Industry, Ministry of Health and Welfare, Doum 4-ro, Sejong-si 339-012, Republic of Korea; ^3^Graduate School of Public Health, Seoul National University, Sillim-dong, Kwanak-gu, Seoul 151-742, Republic of Korea

## Abstract

As the role of traditional medicine in community health improvement increases, a comprehensive health care program for infectious diseases management in child-care centers by Korean medicine doctors was developed. The purpose of this study is to evaluate the effects of the program intervention on infection-related medical care utilization among children. The study used a quasi-experimental design with nonequivalent control group, comparing pre- and post-intervention data of the same children. The program implemented interventions in terms of management, education, and medical examination for the teachers, parents, and children in 12-week period. The frequency of utilization, cost, and prescription days of drugs and antibiotics due to infectious diseases prior to the intervention were compared with those during the 3-month intervention, using health insurance claim data. A panel analysis was also conducted to support the findings. A significant reduction (12%) in infection-related visit days of hospitals was observed with the intervention (incident rate ratio = 0.88, *P* = 0.01). And medical cost, drug prescription days, and antibiotics prescription days were decreased, although not statistically significant. A further cost-effectiveness analysis in terms of social perspectives, considering the opportunity costs for guardians to take children to medical institutions, would be needed.

## 1. Introduction

The number of infants supported in child-care centers has been increasing remarkably, due to changes in major social and demographic trends, altering the structure and function of families in Korea. Parents as well as health care professionals are concerned about the health and safety of children who spend all or part of their days in child-care settings. These concerns include the high incidence of infectious diseases transmission among child-care populations [1]. In fact, it was reported that children who attend child-care centers have higher prevalence of infectious diseases [2–7]. Conventional health intervention program to reduce these incidences of infectious diseases in child-care center seems insufficient.

World Health Organization (WHO) emphasized that traditional medicine can be a useful approach to resolve community health problems [8] and recommended the use of traditional medicine in primary health care at policy level [9]. In Korea, it is reported that 77.5% of Korean people have used Korean traditional medicine. Furthermore, 94.3% of Korean traditional medical care is provided in ambulatory settings such as in primary health care [10]. Such results show the important role of Korean medicine in both protecting and restoring health, which are appropriate for the enhancement of primary health.

Although the public health program of Korean medicine through public health care centers was implemented since 2005 [11], comprehensive health care program in child-care centers has not been yet implemented. This has motivated us to develop a comprehensive health care program for infectious diseases management in child-care centers by Korean medicine doctors. In this study, we conducted the evaluation of the program effect on medical care utilization to prevent infectious diseases and to promote health, using data from Korea Health Insurance Review and Assessment service (HIRA), following prior study using survey data from parents of children and teachers [12].

## 2. Materials and Methods

### 2.1. Study Design and Subjects

The study adopts a quasi-experimental design with nonequivalent control groups and measures, taking pre- and post-intervention evaluation of the same children. This design is considered to be useful for community-based interventions, in which randomization is not possible. First, six day-care centers located in Seoul and Kyung-gi province volunteered for this program. Researchers visited there and explained the purpose and contents of the program and then collected data with the permission of the parents of children. To control Hawthorn effect, participants were not informed of the program evaluation criteria. Second, other six day-care centers, which shared similar characteristics in size, location and ownership, have been selected as control groups. To control for the contamination of control group (John Henry effect), the purpose of the research is explained to examine the current state of infectious diseases prevalence. Third, six doctors of Korean medicine were provided with common education about the program and assigned to each of the experimental group.

After 3 months of implementation, we analyzed the claims data of infants to determine the changes in medical care utilization, such as frequency, cost and prescription days of drug and antibiotics between the treated and the untreated. Finally, 227 out of 251 in the treated group and 92 out of 321 in the untreated group were included by informed consent.

### 2.2. Intervention Program

The program provides management, education, and medical examination support for the teachers, parents, and children for 12 weeks (from August 2012 to October 2012). The program was implemented by Korean medicine doctors using preventive concepts of Korean medicine, Yangsaeng (養生) (Yangsaeng (養生) is a traditional health care regimen for the promotion of health and prevention of illness by means of specific principles and methods, whose purpose was to improve longevity and healthy life [13]) and Chimibyeong (治未病) (Chimibyeong (治未病) is a theory to prevent disease from occurring, worsening, being delivered, and so on; in many books, the word Chimibyeong was used as a term of preventing a disease or used as a word meaning treating a disease in the early stage [14]), in cooperation with the parents, children, and teachers. Six Korean medicine doctors were recruited through an online notification in the homepage of “The Association of Korean Medicine,” who were restricted to a pediatrician of Korean medicine, a Korean medicine doctor in Korean medical clinic for pediatric specialty, or Korean medicine doctors with over-5-year clinical experience.

**Figure 1:**
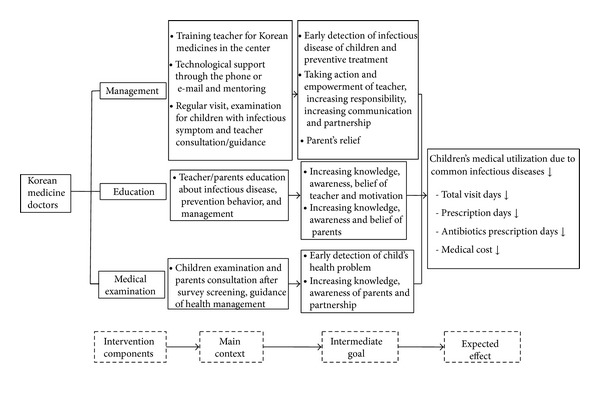
Design of the comprehensive health care program for child-care centers.

The primary goal of the program is to prevent infectious diseases and promote health in child-care centers. The program was supported with inputs from 1 public health professor, 1 professor of pediatrics in the Korean Medicine Hospital of Kyunghee University, and 1 pediatrician for Korean medicine. Specific details of the program are illustrated and summarized in Figure 1.

#### 2.2.1. Management

The management includes the control of infectious diseases in child-care centers. Before intervention, child-care centers were provided with a box of household medicines, including Korean medicines, which treat early stage of common infectious diseases and prevent the progress of diseases. The Korean medicine doctor visits child-care centers in one-on-one setting, educating teachers about the use of household medicines, provided to manage symptoms according to the “Yangsaeng.” The purpose of this visit and education was to improve the knowledge of caregivers and prevent possible contraction of disease through appropriate action from teachers.

To promote a preventive attitude of teachers on infection, teachers were advised to monitor and record the occurrence of symptoms of infectious diseases and children's attendance. When needed, they were guided to use household medicines under the management of doctors with parent's approval. The Korean medicine doctor visits child-care center monthly and follows up observations on children with symptoms by checking the records taken by teachers and through talks with the teachers. In addition, during their visit to the child-care center, the Korean medicine doctors guided the teachers in managing symptoms using household medicines and conducted examination and consultation of children who have infectious symptoms. During the study, the Korean medicine doctor kept in touch with child-care centers through phone and emails, providing consultations and support in emergency cases and in guiding the management of early signs of disease and its symptoms.

#### 2.2.2. Education

Education about a summary of frequent infectious diseases and preventive behavior and management in Korean medicine was provided to parents and teachers once for 80 minutes by Korean medicine doctors.

#### 2.2.3. Medical Examination

The survey was conducted to examine the children's functional and physical symptoms and health status through parent's interviews. The survey consists of systemic questions about the general development of children under the Five Organs theory in Korean medicine. The Korean medicine doctor checks the survey, sorts children who needed consultation and management, and examines them personally including children's health management guidance such as Yangsaeng for their parents and volunteers.

### 2.3. Data and Statistical Analysis

Claim data of 319 infants in agreement was retrieved from Korea “Health Insurance Review and Assessment Service.” Medical care utilization, such as frequency of visit, costs, and prescription days of drugs and antibiotics due to infectious diseases prior to the intervention (from May 1, 2012, to July 31, 2012), was compared with those within the 3 months of intervention (from August 1, 2012, to October 31, 2012). We have also carried out the survey before intervention targeting the full sample (572 infant) to find out the basic features of participants and to compare them with those who do not participate in the study.

We conducted a panel analysis to estimate the effect of the comprehensive health care program on medical care utilization of children, specifically on common infectious diseases. In the case of panel data, the change in medical care utilization by the same individual is traced, so that more exact effect of intervention can be measured. In other words, it is possible to control for individual-specific, time-invariant, and unobserved heterogeneity, which may be correlated with other explanatory variables [15]. Although there are potentially many factors affecting the frequency of infections even when the groups are assessed during the same temporal period, panel model seems to control some features that are hard to observe but can be related to medical care utilization, such as unclean environment, vulnerabilities to infection, related past history of illness that parents may not remember, and inherited traits.

This research also adopted a random effect model, since the study period is too short (3 months) to produce sufficient within-unit variance and has data limitations as it only has two waves. Data was analyzed using STATA ver. 11.

### 2.4. Research Ethics

This research was approved by the Institutional Review Board of the Graduate School of Public Health, Seoul National University (IRB number 19-2012-06-04).

## 3. Results

### 3.1. Group Equivalence

#### 3.1.1. Between the Treated and the Untreated

In the case of the community-based health promotion program, it is difficult to conduct randomization. Since this program participation was voluntary, we matched the case group (one to one) with the control group with similar characteristics in size, location, ownership, and so forth (Table 1).

#### 3.1.2. Between Consenters and Nonconsenters

Since the claim data contain information about individual health and behavior, some participants are concerned about making their medical care utilization information public. Some of the parents did not agree to use information on their children's medical care utilization, and this was more distinct in the untreated group where there was little room for confidence formed between the parents and the researchers. If there is selection bias due to the consent of information, the program effect would be overestimated or underestimated; therefore the first step in the program was to assess homogeneity between consenters and nonconsenters. Before intervention, we carried out survey targeting the full sample (572 infant) to find out the basic characteristics and usual symptoms related to infection and compared the consenters with nonconsenters. This procedure will determine the existence of any systemic difference between consenters and nonconsenters.

In the presurvey, no significant differences between consenters and nonconsenters were detected, except for health insurance coverage (Table 2). National health insurance subscribers are 96.86% in consenters and 91.63% in nonconsenters. Medical care utilization pattern may show differences between national health insurance subscribers and medical aid recipients, but this is adjusted in the panel regression analysis.

In infection-related symptoms, most of them occurred less frequently in consenters. Only nasal discharge was reported more frequently in consenters (Table 3).

### 3.2. Screening of Infection-Related Medical Care Utilization

The medical care utilization data for frequent infectious diseases was screened through a consultation with 1 pediatrician of western medicine and 1 pediatrician of Korean medicine, to select appropriate medical care utilization data for the purpose of this study. In the total of 4,379 cases, 764 cases were excluded such as dental clinic utilization, resulting in 3,615 cases for the analysis. The criteria on disease classification are shown in Table 4. 1,866 cases in 3,615 cases have occurred in the 3 months prior to the intervention, and 1,749 cases have occurred during the intervention which lasted for 3 months.

### 3.3. Basic Characteristics of Participants

Table 5 summarizes the initial demographic characteristics of 319 informants who are divided into treated and untreated groups. Before evaluating the impact of intervention, it was necessary to show that two groups were similar on variables that could be associated with outcomes. As shown in Table 4, no significant differences were detected in dependent variables of two groups. Also, very few differences between two groups were found in independent variables. Higher extent of mother as a main carer of the infant and lower level of household income were observed in the treated group.

### 3.4. The Program Effect on Medical Care Utilization

Medical care utilization was divided into medical care utilization days and total medical cost. Medical care utilization days were subcategorized into total visit days, prescription days of drugs, and prescription days of antibiotics.

Without control for any covariates, total visit days during the intervention period increased by 0.22 in the untreated group and decreased by 0.6 in the treated group. Drug prescription days increased by 0.33 days in the untreated but decreased by 1.12 days in the treated group. Antibiotics prescription days during the intervention period decreased by 0.04 days in the untreated and also decreased by 0.91 days in the treated group. Medical cost increased by W7,863 in the untreated and decreased by W8,757 in the treated group.

In the panel regression, the count data such as visit days, drugs prescription days, and antibiotics prescription days are analyzed by the negative binomial model. First, incidence rate ratios comparing the treated and untreated groups with regard to the medical care utilization days were examined while holding constant the previously discussed covariates. 12% of significant reduction in total visit days of hospitals were associated with the intervention (incident rate ratio = 0.88, *P* = 0.01), but the reductions of drug prescription days and antibiotics prescription days were not statistically significant.

Second, we compared the medical cost due to common infectious diseases between the treated and untreated groups. The medical cost was log-transformed since it is skewed with long right tail. The results show that the intervention program had a negative impact on medical cost, but it was not statistically significant (Table 6).

As a result, total visit days were 2.67 in the treated group and 2.79 in the untreated group. The marginal effect of the program was −0.12.

## 4. Conclusions

It is generally agreed that implementation of comprehensive health care program in child-care centers is important in minimizing common infections and promoting children's health [16–22]. However, documentation of the benefits of such a program has been incomplete. Several leading researches have studied the effect of health intervention program on infections in child-care centers and emphasized the importance of prevention and management for infectious diseases in child-care centers. But some research compared only pre- and post-results without control groups [17, 23], and some just used staff's records or surveys of parents as outcomes [17–22].

Our study tried to overcome these limitations in several ways. First, we performed this study as quasi-experimental design by matching the case group with the control group having similar features like location, ownership, and size. Although the perfect randomization was not possible, the maturation effect, which means changes in characteristics of target population by the passage of time, and extraneous environmental effect could be controlled. Second, we tried to estimate more accurate effect of intervention statistically. The various, observable characteristics of participants were controlled by a multivariate regression model. In addition, time-invariant, unobserved, individual-specific effect that could affect the outcome could be controlled by using a panel model. Third, we used objective data of health insurance claim to measure outcome variables. Analyzing claim data has many advantages, including a lower risk of recall bias and coverage of the whole intervention period into analysis without recall bias, compared to survey. Besides, claim data can provide accurate diagnosis, number of days, and cost due to infections, which makes it possible to screen specific episodes adequate for the study. It is meaningful that this study has overcome some methodological concerns, such as limitation of data supporting the efficacy of the intervention, objective measurements, and analytic techniques, which were mentioned by Huskins [24].

Furthermore, our program tried to take advantages of comprehensive and preventive aspects of traditional medicine and applied it to practice. For example, this program not only provided the health management of children, but also educated teachers and parents about the prevention of common infectious diseases, including health promotion in Korean medicine. So, the intervention encompasses not only primary prevention, but also secondary prevention by providing household polyherbal medicines and conducting medical examination, which permit early detection of diseases and proper reaction.

Empirical results showed that 12% of significant reduction in total visit days of hospitals were associated with the intervention (incident rate ratio = 0.88, *P* = 0.01). This implies that the treated group visited physicians less, specifically 0.12 times less per person, controlling for observable covariates and unobservable time-invariant variables. This is an improved result compared with Park et al. [12], which evaluated the same intervention based on survey and showed that the total medical care utilization due to infectious diseases decreased, but without statistical significance. On the other hand, drugs prescription days, antibiotics prescription days, and medical cost decreased, but not statistically significant.

In this research, we only focused on measuring the effect of child-care center program on medical care utilization due to infectious diseases. If we just focus on the medical utilization, it would not be surprising that providing doctors on-site would decrease the number of visits to outside medical practitioners. But one should note that most of preventive interventions were performed using Korean traditional herbal medicines according to the Korean traditional medicine theory. In addition, this result was just one aspect of evaluation and other comprehensive effects were also reported by Park et al. [12], which showed the significant decrease in children's absence/lateness days and high parents' satisfaction with this intervention.

Finally, this research shows that comprehensive health care program using traditional medicine decreased medical utilization significantly, which can be a big benefit for those parents who work. Reduced medical care utilization of children results in reduced wage loss for working parents, who should accompany their children in case of medical care utilization. Further cost-effectiveness analysis in terms of social perspectives considering the opportunity costs for guardians to take children to the medical institutions would increase the value of the intervention in this study.

Nevertheless, there are also limitations in this study. First, non-randomly assigned control group could pose a selection bias problem although various efforts were made to overcome this limitation. Second, nonconsenters for using their claim data were excluded in data analysis. We compared the demographic, socioeconomic features and frequency of infection-related symptoms between the consenters and nonconsenters and could not observe any systemic differences. But there is still a possibility that it had an effect on the result. Third, we controlled the time-invariant and unobservable confounding factors by panel model, but the individual-specific time-variant factors still could not be controlled completely.

In spite of limitations, this research suggests that comprehensive health care program using traditional medicine can be effective to manage infections in child-care centers. In future studies, the implementation of sufficient long-term intervention to a larger sample would be helpful to overcome the limitations, as the period of this intervention was relatively short and the number of infants that provide information on medical care utilization was not large.

## Figures and Tables

**Table 1:** The characteristics of child-care centers.

Group	Treated						Untreated					
Area	S	S	S	G	G	G	S	S	S	G	G	G
Types	P	PS	PS	Pt	P	K	P	PS	PS	Pt	P	K
Size (number of children)	86	39	31	104	91	133	80	51	39	125	70	160
Number of teachers	12	5	5	9	7	9	15	10	7	11	6	8
Visiting health care profession	N	N	N	N	N	N	N	N	N	N	N	N
Health record	Y	Y	Y	Y	Y	Y	Y	Y	Y	Y	Y	Y

S: Seoul, G: Gyeonggi-do.

P: public; PS: private (Seoul-type); Pt: private; K: kindergarten.

N: no; Y: yes.

**Table 2:** Characteristics of infants.

		Consenters (%) (*n* = 319)	Nonconsenters (%) (*n* = 253)	*P*
Age		5.52 ± 1.20	5.67 ± 1.28	0.14

Sex	Male	181 (56.74)	116 (48.74)	0.06
	Female	138 (43.36)	122 (51.26)	

Duration	Under 1 yr	81 (25.47)	48 (19.05)	0.07
	Over 1 yr	237 (74.53)	204 (80.95)	

Past history	Yes	168 (57.14)	143 (57.66)	0.90
	No	126 (42.86)	105 (42.34)	

Number of siblings		1.97 ± 0.64	2.05 ± 0.68	0.17

Main carer	Mother	271 (89.44)	216 (86.06)	0.22
	etc.	32 (10.56)	35 (13.94)	

Health insurance coverage	Medicaid	9 (3.14)	20 (8.37)	0.01∗
	National Health Ins.	278 (96.86)	219 (91.63)	

Household income	Under 4,000	162 (54.92)	136 (59.13)	0.33
	Over 4,000	133 (45.08)	94 (40.87)	

Mom's education	Below high school	53 (18.15)	38 (15.70)	0.45
	Over college	239 (81.85)	204 (84.30)	

Mom's job	Working mom	158 (53.02)	135 (55.79)	0.52
	Household	140 (46.98)	107 (44.21)	

*P < 0.05.

**Table 3:** Infection-related symptoms during 2 weeks, shortly before the intervention.

Characteristics or variables	*n* = **572**			
	Consenters (*n* = 319)	Nonconsenters (*n* = 253)	*χ* ^2^	*P*
Fever				
Yes	14.85%	14.29%	0.04	0.85
No	85.15%	85.71%		
Cough				
Yes	28.38%	29.37%	0.93	0.63
No	71.62%	70.63%		
Sneeze				
Yes	14.19%	15.14%	0.10	0.75
No	85.81%	84.86%		
Nasal discharge				
Yes	40.59%	30.95%	5.54	0.02∗
No	59.41%	69.05%		
Nasal congestion				
Yes	20.79%	20.63%	0.00	0.96
No	79.21%	79.37%		
Epistaxis				
Yes	11.55%	10.71%	0.10	0.76
No	88.45%	89.29%		
Abd. pain				
Yes	15.51%	13.89%	0.29	0.59
No	84.49%	86.11%		
Diarrhea				
Yes	7.59%	5.56%	0.92	0.34
No	92.41%	94.44%		
Vomiting				
Yes	3.63%	2.78%	0.32	0.57
No	96.37%	97.22%		
Ear pain				
Yes	3.30%	1.98%	0.91	0.34
No	96.70%	98.02%		
Ear ooze				
Yes	0.66%	0.00%	1.67	0.20
No	99.34%	100.00%		
Eye itch				
Yes	6.93%	5.16%	0.75	0.39
No	93.07%	94.84%		
Bloodshot eyes				
Yes	4.62%	3.97%	0.14	0.71
No	95.38%	96.03%		
Eye discharge				
Yes	9.24%	5.56%	2.67	0.10
No	90.76%	94.44%		
No symptom				
Yes	0.66%	0.00%	1.67	0.20
No	99.34%	100.00%		

*P < 0.01.

**Table 4:** Inclusion and exclusion criteria of diagnosis.

Classification of diseases	Inclusion criteria	Exclusion criteria	Total episodes	Included episodes
Certain infectious and parasitic diseases (A00-B99)	Including all		201	201
Neoplasms (C00-D48)		Excluding all	0	0
Diseases of the blood and blood-forming organs and certain disorders involving the immune mechanism (D50-D89)		Excluding all	1	0
Endocrine, nutritional, and metabolic diseases (E00-E90)		Excluding all	4	0
Mental and behavioral disorders (F00-F99)		Excluding all	113	0
Diseases of the nervous system (G00-G99)		Excluding all	58	0
Diseases of the eye and adnexa (H00-H59)		Excluding myopia, strabismus, and so forth	112	87
Diseases of the ear and mastoid process (H60-H95)		Excluding cholesteatoma and so forth	269	248
Diseases of the circulatory system (I00-I99)		Excluding all	0	0
Diseases of the respiratory system (J00-J99)	Including all		2840	2840
Diseases of the digestive system (K00-K93)		Excluding dental diseases, hernia, etc.	275	186
Diseases of the skin and subcutaneous tissue (L00-L99)		Excluding all	160	0
Diseases of the musculoskeletal system and connective tissues (M00-M99)		Excluding all	12	0
Diseases of the genitourinary system (N00-N99)	Including all		15	15
Pregnancy, childbirth, and the puerperium (O00-O99)		Excluding all	0	0
Certain conditions originating in the perinatal period (P00-P96)		Excluding all	0	0
Congenital malformations, deformations, and chromosomal abnormalities (Q00-Q99)		Excluding all	14	0
Symptoms, signs, and abnormal clinical and laboratory findings (R00-R99)	Including epistaxis, cough, fever, and abd. pain	Excluding abnormalities of gait and mobility, lack of growth, and so forth	143	33
Injury, poisoning, and other certain consequences of external causes (S00-T98)		Excluding all	129	0
External causes of morbidity and mortality (V01-Y98)		Excluding all	0	0
Factors influencing health status and contact with health services (Z00-Z99)		Excluding all	10	0
Codes for special purposes (U00-U99)∗	Including pattern/syndrome of lung (肺系)	Excluding fatigue due to overexertion (勞倦)	24	6

Total			4379	3615

*Including medical care utilization of traditional Korean medicine.

**Table 5:** Characteristics of participants.

		Treated (*n* = 227)	Untreated (*n* = 92)
Dependent variables			

Medical care utilization	Visit days	6.01 ± 0.41	5.52 ± 0.52
	Total cost (W)	87,640 ± 8,938	67,873 ± 6,936
	Prescription days	16.22 ± 1.13	17.37 ± 1.89
	Prescription days of antibiotics	10.80 ± 0.91	11.12 ± 1.24

Independent variables			

Age		5.48 ± 0.08	5.62 ± 0.12

Sex	Male	131 (57.71)	50 (54.35)
	Female	96 (42.29)	42 (45.65)

Duration	Under 1 yr	63 (28.90)	18 (21.69)
	Over 1 yr	155 (71.10)	65 (78.31)

Past history	Yes	121 (56.81)	47 (58.02)
	No	92 (43.19)	34 (41.98)

Number of siblings		1.96 ± 0.61	1.98 ± 0.71

Main carer∗	Mother	202 (91.82)	69 (83.13)
	etc.	18 (8.18)	14 (16.87)

NHI	Medicaid	9 (4.04)	3 (3.30)
	National Health Ins.	214 (95.96)	88 (96.70)

Income∗	Under 4,000	128 (60.38)	34 (40.96)
	Over 4,000	84 (39.62)	49 (59.04)

Mom's education	Below high school	36 (16.98)	17 (21.25)
	Over college	176 (83.02)	63 (78.75)

Mom's Job	Working mom	103 (47.91)	37 (44.58)
	Household	112 (53.02)	46 (55.42)

*P < 0.05.

**Table 6:** The effect of comprehensive health care program on medical care utilization.

		Total visit days			Prescription days			Antibiotics prescription days			Medical cost		
		IRR∗∗	Std. err.	*P* > *z*	IRR∗∗	Std. err.	*P* > *z*	IRR∗∗	Std. err.	*P* > *z*	Coef.	Std. err.	*P* > *z*
Program (ref. untreated)	Treated	0.88∗	0.04	0.01	0.91	0.06	0.14	0.90	0.08	0.24	−0.30	0.29	0.30

Age (ref. under 4)	Over 5	0.81∗	0.04	0.00	0.75∗	0.04	0.00	0.79∗	0.04	0.00	−0.59∗	0.23	0.01
Sex (ref. male)	Female	0.87	0.09	0.21	0.94	0.10	0.60	0.91	0.09	0.33	−0.26	0.48	0.59
Past history (ref. no)	Yes	1.59∗	0.18	0.00	1.37∗	0.15	0.01	1.48∗	0.15	0.00	0.92	0.48	0.06
Duration (ref. under 1 yr)	Over 1 yr	0.86	0.13	0.32	1.07	0.16	0.65	1.07	0.14	0.62	0.48	0.64	0.45
Sibling (ref. no)	Yes	0.51	0.42	0.41	0.38	0.31	0.23	0.41	0.27	0.18	−3.23	3.92	0.41

Main carer (ref. ect.)	Mother	0.61∗	0.11	0.01	0.70	0.13	0.06	0.65∗	0.10	0.01	−1.99∗	0.82	0.02
job of mother (ref. non)	Working	1.20	0.14	0.12	1.23	0.15	0.09	1.07	0.12	0.50	0.64	0.51	0.21

Medical aid (ref. Medicaid)	NHI	0.66	0.17	0.12	0.59	0.16	0.05	0.70	0.15	0.10	−1.45	1.20	0.23
Education of mom (ref. under high school)	Over college	0.93	0.14	0.61	0.86	0.13	0.31	0.72∗	0.09	0.01	−0.57	0.65	0.38
Household income (ref. under 400)	Over 400	0.97	0.11	0.76	0.98	0.12	0.85	0.94	0.10	0.59	−0.16	0.50	0.76

_cons											18.55∗	4.34	0.00

LR test		0.00			0.00			0.01					
Log likelihood		−1,417.63			−2,026.76			−1,762.01					

*P < 0.05.

**Incidence rata ratio.
